# Terminal‐Matched Topological Photonic Substrate‐Integrated Waveguides and Antennas for Microwave Systems

**DOI:** 10.1002/advs.202404163

**Published:** 2024-07-04

**Authors:** Zhixia Xu, Xiaonan Sun, Haotian Wu, Zengxu Xiong, Xue Zhou, Haoxi Yu, Xiaoxing Yin, Daniel F. Sievenpiper, Tie Jun Cui

**Affiliations:** ^1^ State Key Laboratory of Millimeter Waves Southeast University Nanjing 210096 China; ^2^ School of Information Science and Technology Dalian Maritime University Dalian 116026 China; ^3^ School of Electrical and Electronic Engineering Nanyang Technological University Singapore 639798 Singapore; ^4^ Electrical and Computer Engineering Department University of California San Diego San Diego CA 92093 USA

**Keywords:** edge state, leaky‐wave antenna, substrate integrated waveguide, topological photonic crystal, wireless communication

## Abstract

In engineered photonic lattices, topological photonic (TP) modes present a promising avenue for designing waveguides with suppressed backscattering. However, the integration of the TP modes in electromagnetic systems has faced longstanding challenges. The primary obstacle is the insufficient development of high‐efficiency coupling technologies between the TP modes and the conventional transmission modes. This dilemma leads to significant scattering at waveguide terminals when attempting to connect the TP waveguides with other waveguides. In this study, a topological photonic substrate‐integrated waveguide (TPSIW) is proposed that can seamlessly integrate into traditional microstrip line systems. It successfully addresses the matching problem and demonstrates efficient coupling of both even and odd TP modes with the quasi‐transverse electromagnetic modes of microstrip lines, resulting in minimal energy losses. In addition, topological leaky states are introduced through designed slots on the TPSIW top surface. These slots enable the creation of TP leaky‐wave antennas with beam steering capabilities. A wireless link based on TPSIWs are further established that enables the transmission of distinct signals toward different directions. This work is an important step toward the integration of TP modes in microwave systems, unlocking the possibilities for the development of high‐performance wireless devices.

## Graphical abstract

Topological photonic substrate‐integrated waveguide is seamlessly integrated into the traditional microstrip line systems, achieving efficient coupling of both even and odd TP modes with the quasi‐transverse electromagnetic modes of the microstrip lines. This breakthrough minimizes energy losses and opens new possibilities for the practical application of topological photonics in microwave systems, inspiring new transmission lines and antenna designs.

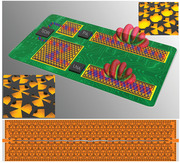

## 1 Introduction

The field of photonics has experienced a significant evolution with the emergence of photonic topological insulators (PTIs), which mimic the behavior of quantum spin hall effects.^[^ ^1^ ^]^ The PTIs revolutionize transmission by providing immunity to backscattering,^[^ ^2^ ^]^ and are called as topological photonic (TP) modes.^[^ ^3^, ^4^ ^]^ Various forms of TP modes have been investigated extensively,^[^ ^5^, ^6^, ^7^ ^]^ such as quantum Hall TP modes based on gyromagnetic materials,^[^ ^8^, ^9^ ^]^ valley TP modes based on *C*_3v_ symmetry,^[^ ^10^, ^11^, ^12^ ^]^ and quantum spin Hall TP modes based on the hybridization of transverse electric or transverse magnetic modes.^[^ ^13^, ^14^ ^]^ The potential of TP modes has led to fantastic applications,^[^ ^15^, ^16^ ^]^ including high‐performance transmission,^[^ ^17^, ^18^, ^19^ ^]^ digital coding devices,^[^ ^20^, ^21^ ^]^ programmable.^[^ ^22^ ^]^ and wireless chips,^[^ ^23^ ^]^ and chiral‐sorting unidirectional transmissions.^[^ ^24^, ^25^ ^]^

Despite the promising attributes of the TP modes, their integration into traditional systems poses a considerable challenge due to substantial differences from the conventional transmission modes. While unidirectional transmission characteristics.^[^ ^26^, ^27^ ^]^ and high‐quality cavity designs.^[^ ^28^, ^29^, ^30^ ^]^ have been showcased, their practical applications face impediments in the form of strong backscattering at connection points. In the optical band, the integration of dielectric waveguides into PTI slabs has shown promise in achieving a seamless transition.^[^ ^31^, ^32^ ^]^ However, translating this success to the matching between TP modes and the 50 Ω terminal characteristic impedance of the traditional microwave transmission lines, a standard in common microwave systems, remains a gap in the current research.

An existing report details the matching of a slot line to a dual‐layered PTI structure,^[^ ^33^ ^]^ but the resulting structure supports a mixed mode, combining the quasi‐transverse‐electromagnetic (QTEM) mode and the TP mode, leading to inherent radiation loss and electromagnetic compatibility issues due to its open structure. Furthermore, the integration technology of TP antennas is not yet mature. The most generic form of TP antenna is the horn antenna. When unidirectional TP modes reaches at the terminal of the waveguides, the energy would be totally radiated.^[^ ^34^, ^35^ ^]^ However, these horn TP antennas could not steer the radiation beam, and it is meaningful to explore leaky‐wave antenna (LWA) designs based on the TP modes. This area is still limited to the conceptual simulations,^[^ ^36^, ^37^ ^]^ lacking experimental realization.

In this study, we address these challenges by realizing the valley TP modes in substrate‐integrated waveguides (SIWs), termed as TPSIWs, and propose a methodology to achieve terminal matching between even and odd TP modes of TPSIWs with the QTEM modes of the typical 50 Ω microstrip lines. This advancement allows for the direct integration of TPSIWs into the common microwave systems, overcoming the impedance mismatch hurdle. Moreover, the design of two distinct types of TP leaky‐wave antennas (TPLWAs) based on the even and odd modes, achieved by strategically cutting transverse or longitudinal slots on the top layer of TPSIWs, enables forward and backward beam steering capabilities. The novelty and importance of this work lies in the comprehensive realization of TPSIWs and the provision of a practical solution for integrating the TP modes into microwave systems, enhancing the potential for advanced microwave system designs and wireless applications.

## 2 Results

### 2.1 Integration of the TPSIW Devices

**Figure 1:**
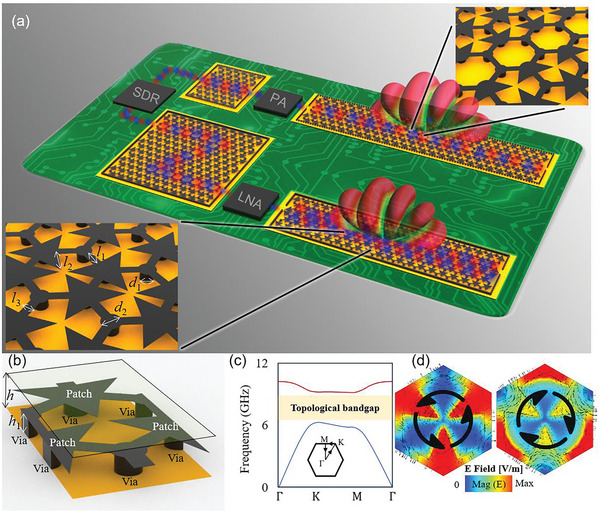
Integration of the TPSIWs in microwave systems. a) The TPSIWs are used to connect various integrated circuits with ideal matching performance and can also work as antennas. b) The unit cell structure consists of two substrates (RO 4350B) and three 0.035‐mm‐thick metallic layers (up layer, middle patch, ground plane). The geometric parameters are: *l*_1_ =  2.6 *mm*,   *l*_2_ =  4.54 *mm*,   *l*_3_ =  1.5 *mm*,    *d*_1_ =  2 *mm*,   *d*_2_ =  2.6 *mm*,  *h*  =  2.032 *mm*,  *h*_1_ =  1.524 *mm*. c) The band diagram. d) Eigen field distributions with the C*_3v_* symmetry at the valley point where the energy is confined at the triangular patches in the lower band, and the energy is confined at the circular patches in the upper band. The marked Poynting vector distribution shows the opposite chirality.

**Figure** **1**a illustrates the application of the TPSIW devices in microwave circuits, where the TPSIWs can connect different integrated chips smoothly. In the current stage, various integrated chips are developed with a typical 50 Ω terminal impedance, supporting the QTEM mode; therefore, it requires that TPSIW devices achieve 50 Ω terminal impedance to avoid any refection at terminals. Recent reports have highlighted the emergence of several multilayer TP structures based on the printed‐circuit‐board (PCB) process.^[^ ^38^, ^39^ ^]^ Nevertheless, these structures consistently overlook the critical aspect of terminal matching, posing a significant obstacle to their practical applications. To address the essential issue of achieving terminal matching between the TP modes and common QTEM modes, we introduced a solution by incorporating a pair of slots in the top layer to facilitate the insertion of a microstrip line into the TPSIWs. Given the distinct field distributions of even and odd TP modes, we devised two distinct matching technologies tailored to each. Moreover, we report the LWA designs based on the TPSIWs.

### 2.2 Unit Cells and Eigen‐Mode Analyses

Figure 1b illustrates the composition of the proposed TPSIW unit cell. This innovative design comprises a multilayer structure composed of two substrates and three copper layers. The six vertices of each honeycomb unit are interconnected with the ground plane via metallic vias, while neighboring blind vias are linked by metallic strips. Within this configuration, a substrate separates the patches from a metallic top plane. The band diagram of the unit cell, depicted in Figure 1c, highlights a distinct topological bandgap emerging ≈8 GHz. The eigen field distributions at the K points display *C*_3v_ symmetry, showcased in Figure 1d. Those vias connecting the metasurface with the ground plane are crucial to suppress the TE‐polarized waveguide modes and support a pure TP edge state. The comparison between the structures with and without vias is discussed (Figures S1, S2, and S3, Supporting Information).

**Figure 2:**
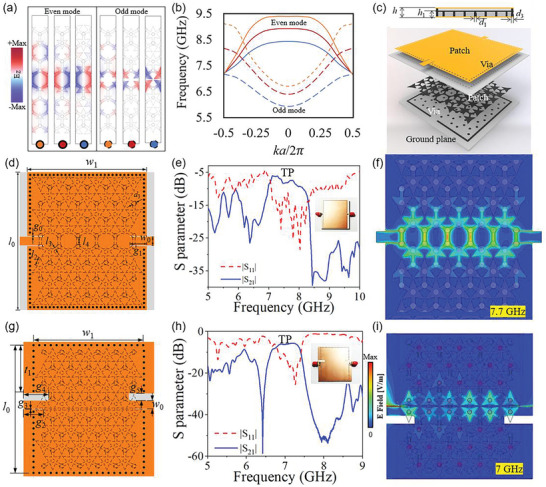
Eigenmode analysis of TP edge states and the measured performance of TPSIWs. a) Parameter sweeping analysis: side length *l*_2_ = 4.54 mm (blue), *l*_2_ = 3.41 mm (red), *l*_2_ = 2.27 mm (orange). Field distributions of the even and odd TP modes at the interface. b) Dispersion curves. c) Configuration of even‐mode TPSIW and the cross‐section view. d) The top view of the even‐mode TPSIW. e) Measured *S* parameters of the even‐mode TPSIW. f) Electric field distributions of the even‐mode TPSIW. g) The top view of the odd‐mode TPSIW. h) Measured *S* parameters of the odd‐mode TPSIW. i) Electric field distributions of the odd‐mode TPSIW. Geometric parameters: *w*_0_ =  4.6 *mm*,   *w*_1_ =  60 *mm*,   *l*_0_ =  70 *mm*,   *l*_4_ =  3.77 *mm*,   *g*_0_ =  0.4 *mm*,   *g*_1_ =  8 *mm*,   *g*_2_ =  6 *mm*,   *g*_3_ =  0.23 *mm*,    *g*_4_ =  9 *mm*,  *g*_5_ =  4 *mm*, *t*_1_ =  26 *mm*,   *d*_3_ =  1 *mm*.

Further investigation involved eigenmode simulations of the superlattice. As shown in **Figure** **2**a, most of the energy is confined along the interface of the two bulk lattices. The observed symmetry in the phase distribution of the electric field led to the identification of two distinct TP modes: the even and odd TP modes, marked by solid and dash lines. In Figure 2b, dispersion curves illustrate that the even mode, found in the higher frequency band, functions as a backward transmission mode, while the odd mode residing in the lower band serves as a forward transmission mode. By tuning the size of the patches, we can adjust the TP passband, which is evident from the dispersion curves. When the size of the triangular patches is decreased, the passbands of both even and odd TP modes shift toward higher frequencies. The odd mode maintains a nearly constant bandwidth, meaning that both high and low cut‐off frequencies shift simultaneously. The low cut‐off frequency of the even mode is fixed ≈7.25 GHz, while the high cut‐off frequency is determined by the size of the triangular patches, shifting from 8.5 to 9.5 GHz. We simulated the corresponding TPSIWs with varying patches (Figures S4, Supporting Information).

### 2.3 Terminal‐Matched TPSIWs

The top layer of the even‐mode TPSIW features symmetrically cut slots, depicted in Figure 2c,d wherein a 50 Ω characteristic impedance microstrip line is precisely inserted at the interface's midpoint. The matching efficiency is notably contingent upon the geometric parameters of the slots and via holes at both terminals, as discussed in the parameter sweeping analysis (Figures S5, S6, and S7 Supporting Information). Subsequently, a prototype of the even‐mode TPSIW was fabricated and measured, illustrated in Figure 2e. Measured results effectively confirm the transmission of the even TP mode, agreeing with the blue solid line in Figure 2b. Moreover, in Figure 2f, the simulated energy distribution of the electric field within the transmission band confirms the successful excitation and transmission of the even TP mode along the structure's interface.

In the configuration of the odd‐mode TPSIW, two slots of different widths are asymmetrically cut on the top of the odd‐mode TPSIW, as illustrated in Figure 2g. The design incorporates a narrow slot positioned at the interface's midpoint, effectively generating anti‐phase electric fields on both sides. This serves the purpose of coupling the odd TP mode from QTEM mode. Additionally, a wider slot is used to enhance and tune the matching performance of the system. Following the optimization, a prototype of the odd‐mode TPSIW was fabricated and measured, represented in Figure 2h. The measured S‐parameters distinctly reveal the presence of the odd TP modes, agreeing with the blue dash line in Figure 2b. Further validation of the ideal transition from QTEM mode to the odd TP mode is provided through simulated electric field distributions within the structures, as shown in Figure 2i.

**Figure 3:**
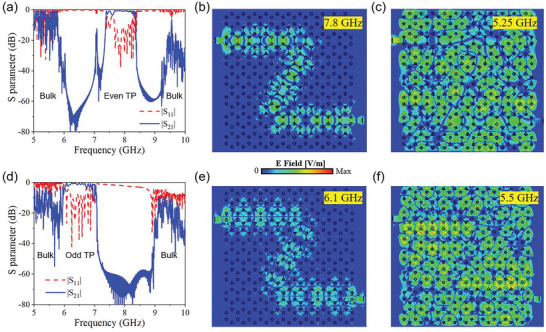
Simulated lossless terminal‐matching TPSIWs with a complex transmission route. a) S parameters of the even‐mode TPSIW. b) Field distribution of lossless even TP transmission. c) Field distribution of bulk states in even‐mode TPSIW. d) S parameters of the odd‐mode TPSIW. e) Field distribution of lossless odd TP transmission. f) Field distribution of Bulk states in odd‐mode TPSIW.

As shown in Figures 2e–h, the reflection efficiency S_11_ is below −15 dB, indicating the ideal matching performance between the microstrip line and the TPSIW. However, the transmission efficiency S_21_ is ≈−5 dB, indicating obvious losses brought by the intrinsic dielectric and Ohmic losses. It is conceivable that we can enhance transmission performance by employing substrates characterized by lower intrinsic losses. To explore this concept further, let us consider the utilization of an ideal dielectric substrate and a perfect conductor. We establish lossless models encompassing complex transmission routes, as illustrated in **Figure** **3**. We confirm that the terminal is still matched to the typical microstrip line of 50 Ω characteristic impedance. The bulk states and perfect TP edge transmission are revealed by both S_21_ and simulated electric field distributions. These results underscore the potential for achieving superior transmission performance of TPSIWs when utilizing substrates and metals with reduced intrinsic losses. The valley topological photonic modes preserve time‐reversal symmetry, and their unidirectional properties have recently been a subject of controversy, as discussed.^[^ ^32^ ^]^ Our structure shows very strong robustness when the height of arbitrary PEC defect is below the via holes (Figure S8, Supporting Information).

### 2.4 Leaky‐Wave Radiation from TPSIWs

**Figure 4:**
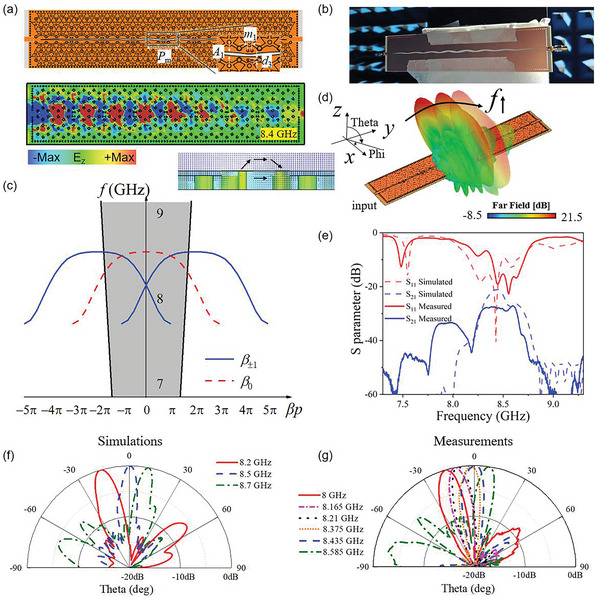
Periodic TPLWA. a) Even‐mode TPLWA with a sinusoidal slot and corresponding simulated electric field distribution at 8.4 GHz. The structural modulation periodic *P*_m_ = 30 mm, the slot width *A*_1_ varies from 1.3 to 2.2 mm, the diameter of via hole *d*_3_ = 1 mm, the distance between via holes *m*_1_ = 2 mm, and the total length of the antenna is 264 mm. b) The fabricated sample placed in the microwave anechoic chamber. c) Dispersion of space harmonics located in the fast‐wave region. d) Simulated beam steering in the 3D space. e) Measured and simulated S‐parameters. f) Simulated radiation pattern in the 2D cross‐section. g) Measured far‐field radiation patterns.

Given the successful development of TPSIWs with excellent terminal performance, the concept of TPLWAs through transmission leakage arises, as shown in **Figure** **4**. The foundational LWA theory remains pertinent in the context of TP modes.^[^ ^40^ ^]^ This identifies the propagation of TP modes into two categories: fast and slow waves based on the relationship between the phase constant of the fundamental TP modes, β_0_, and the free space radiation wavevector, *k*_0_. In the fast‐wave realm (β_0_ < *k*_0_), the fundamental mode inherently generates leakage via quasi‐uniform/uniform slots. Conversely, in the slow‐wave realm (β_0_ > *k*_0_), it is necessary to design periodically modulated slots to expand the fundamental slow‐wave TP mode into a spatial harmonic series expressed as $\beta_{n}=\beta_{0}\;\pm \frac{2\pi n}{P_{m}}$ (*n*  =  0, ±1, ±2, ±3⋅⋅⋅),^[^ ^41^ ^]^ with *P_m_* denoting the structural modulation period. These space harmonics, conforming to the fast‐wave condition (β_*n*_ < *k*_0_), facilitate the generation of leaky‐wave radiation. The direction of the radiation beam θ_*m*_ can be prognosticated as $\theta_{m}=\;arcsin\frac{\beta_{n}}{k_{0}}$.

The even TP mode is confined to the central interface with symmetrically distributed electric fields on both sides and synchronously phased, as shown in Figure 4a. While it seems intuitive to create a longitudinal slot along the midpoint of the even‐mode TPSIW, this structure alone does not generate leaky‐wave radiation from the fundamental mode (Figure S9, Supporting Information). To achieve an expanded beam steering range encompassing the broadside direction, we harness the inherent slow‐wave region of the even TP mode. This is achieved through the design of periodically modulated slots, facilitating the generation of space harmonics. The slot's shape is tailored according to a sinusoidal function, with inserted via holes possessing the same modulation period to enhance matching performance around the open stopband (Figure S10, Supporting Information). This matching approach is common in traditional LWA designs.^[^ ^42^ ^]^ The fabricated sample is shown in Figure 4b. Through the implementation of the periodically modulated slots, we generate space harmonics (β_±1_ < β_0_). The Brillouin diagram for the even TP mode, depicted in Figure 4c, showcases the dispersion of the 1st‐order space harmonic (β_±1_), which show beam steering capability within the fast‐wave region.

Figure 4a presents the simulated model where the electric field distribution shows exponential energy decay along the transmission direction. Furthermore, the insertion picture displays the electric field vector distribution on the cross‐section of the structure. The electric field vector is perpendicular to the slot, resulting in radiation with an electric field polarized along the *x*‐axis. Figure 4b shows a picture of the fabricated antenna in the microwave anechoic chamber. Figure 4c shows the phase constant of the fundamental mode as well as the first‐order spatial harmonic modes. Detailed analysis of the attenuation constant is also presented (Figure S11, Supporting Information). The 3D beam steering performance is presented in Figure 4d. The experimentally retrieved S‐parameters are shown in Figure 4e, where the measured results match well with the simulations. The terminal is still matched to the 50 Ω microstrip line, and simulated and measured normalized radiation patterns are compared in Figure 4f,g. The main beam can scan within a range of 30 degrees, covering the broadside. There is a tiny frequency shift toward lower frequencies of the measured results, and we can confirm that it is caused by the utilized glue when stitching two substrates together. The total radiation efficiency is below 45% because of the high dielectric loss in the substrate (Figures S12 and S13), which can be optimized if we use a better substrate with lower intrinsic loss or the air‐filled SIW technology.^[^ ^43^ ^]^ The open‐stopband phenomenon is still obvious in the lossless model, waiting for further study to overcome.^[^ ^44^ ^]^

**Figure 5:**
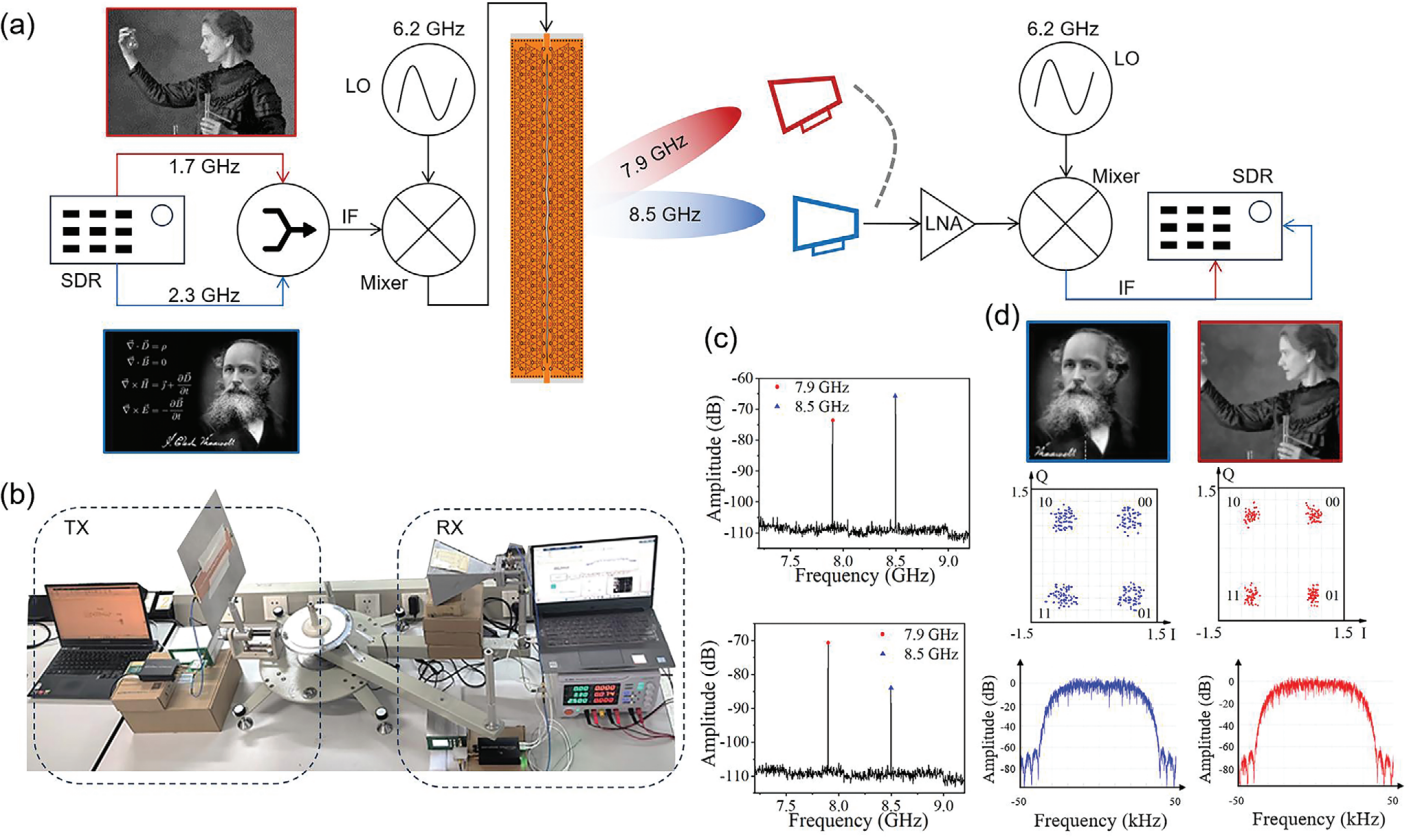
Multi‐channel wireless communications link based on the TPLWA. a) Configurations of the QPSK wireless transmitter and receiver system. b) Photo of the experimental environment. c) Received spectrum from antenna at different angles. d) Received spectrum and demodulated information from antenna at different angles. Based on the Simulink toolbox of MATLAB, the error vector magnitude (EVM) is 13%, the modulation error ratio (MER) is 25.2 dB, and the bitrate is set as 100 kbps.

Recently, it has been reported to apply LWA in the CubeSat communication.^[^ ^45^ ^]^ or conformal‐surface designs.^[^ ^46^ ^]^ besides the beam‐scanning radar applications.^[^ ^47^ ^]^ To evaluate the frequency‐bias beam steering capabilities of the TPLWA, we define a practical application scenario for a multi‐channel wireless communication link using a quadrature phase shift keying (QPSK) scheme. This involves the concurrent transmissions of two distinct waveforms, each carrying unique information, towards disparate users (Figure S14, Supporting Information). **Figure** **5**a,b illustrate the experimental setup, with the transmitter (TX) comprising of the commercial software‐defined radio (SDR) equipment. We transmitted two messages, each associated with a different intermediate frequency (IF) and containing distinct images of Maxwell and Marie Curie. To shift the spectrum to higher frequencies, a mixer was employed, with a local oscillator (LO) frequency set at 6.2 GHz. The TPLWA realized the directional transmission of these waves. On the receiver (RX) end, a horn antenna was positioned to capture the microwaves, allowing for direct spectrum monitoring at various positions, as shown in Figure 5c. Consequently, by observing these variations, we could discern and subsequently demodulate the distinct digital signals, as shown in Figure 5d. This experimental arrangement not only showcases the ability of the TPLWA for frequency‐bias beam steering but also highlights its potential for practical applications in multi‐channel wireless communications.

## 3 Conclusion

Our research reports advancements in the application of topological metamaterials in the microwave band. We first realize high‐efficiency mode matching between topologic edge modes and QTEM mode, making the seamless integration of TPSIWs into conventional 50‐Ω‐impedance microwave systems possible. Furthermore, we propose the concept of the topological LWA by cutting slots on the surface of TPSIW, realizing the beam scanning capability. The dielectric loss is the main drawback waiting for overcome in order to increase the total radiation efficiency. We can further consider the utilization of a lower‐loss substrate or an air‐filled substrate as a possible solution. We also integrate the TPLWA into a microwave wireless system to show the power of multi‐channel communication ability. This research bridges the theoretical concepts of topological metamaterials with device designs. It is foreseeable that more devices with competitive performance can be developed based on our matching technology.

## 4 Experimental Section

The TPSIW and related TPLWAs were designed by Ansys HFSS. The fabricated TPSIW was based on two substrates RO4003C with relative permittivity ≈4 and loss tangent ≈0.0007. The thickness of the two substrates are 0.508 and 1.524 mm, respectively. Eigen mode simulations are based on ANSYS HFSS. Full‐model simulations are based on CST Microwave Studio. The vector network analyzer used for measurements is Agilent N5230A.

## Conflict of Interest

The authors declare no conflict of interest.

## Supporting information

Supporting Information

## Data Availability

The data that support the findings of this study are available from the corresponding author upon reasonable request.
