# Supplementary material for: Terminal‐Matched Topological Photonic Substrate‐Integrated Waveguides and Antennas for Microwave Systems
Source: Adv Sci (Weinh). 2024 Jul 4;11(33):2404163. doi: 10.1002/advs.202404163 (PMC11434011; doi:10.1002/advs.202404163)
Supplement: Supplementary file 1 — Supporting Information [file ADVS-11-2404163-s001.docx]

Supporting Information

Terminal-matched topological photonic substrate-integrated waveguides and antennas for microwave systems

*Zhixia Xu, Xiaonan Sun, Haotian Wu, Zengxu Xiong, Haoxi Yu, Xue Zhou, Xiaoxing Yin, Daniel F. Sievenpiper, and Tie Jun Cui^*^*

Content

[Trivial and nontrivial unit cells with via holes 2](#_Toc169041074)

[Trivial and nontrivial unit cells without via holes 2](#_Toc169041075)

[Parameters affecting topological edge states 3](#_Toc169041076)

[Optimization of the matching performance 5](#_Toc169041077)

[Robustness against PEC defects 6](#_Toc169041078)

[Uniform odd-mode TPLWAs 7](#_Toc169041079)

[Transform of electric vector distributions of even-mode TPLWAs 8](#_Toc169041080)

[Radiation efficiency of even-mode TPLWAs 9](#_Toc169041081)

[Multi-channel wireless link based on TPLWA 11](#_Toc169041082)

# Trivial and nontrivial unit cells with via holes

Figures S1(a) and (b) illustrates the composition of the proposed trivial and nontrivial unit cell, respectively. The *C*_6v_ symmetric trivial structure exhibits a degenerate point around 8 GHz. By comparison, the band diagram of the *C*_3v_ symmetric nontrivial unit cell highlights a distinct topological bandgap emerging around 8 GHz. The generation of the chiral Poynting vector distribution at valley points also show the nontrivial topology of the structure.


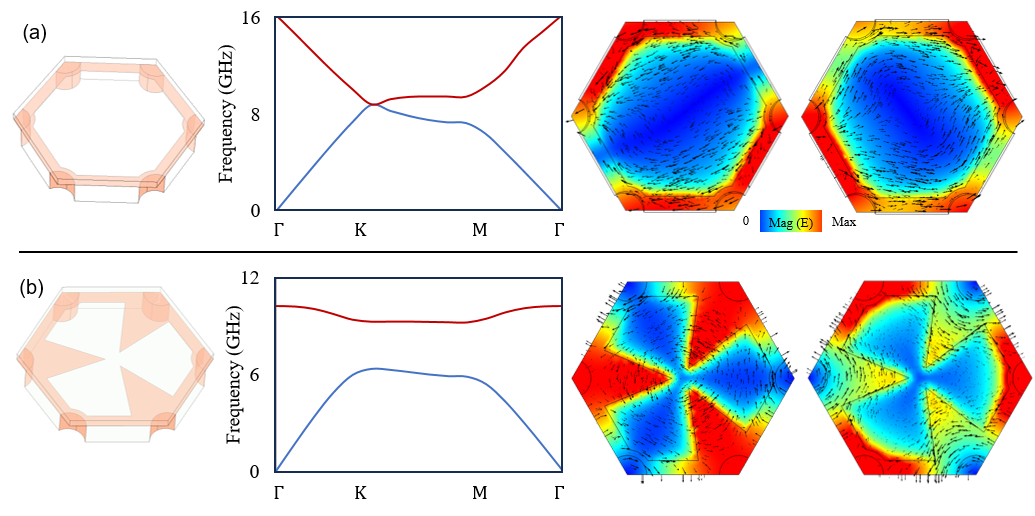


**FIG. S1. Trivial and nontrivial unit cells with via holes.** (a) Trivial unit cell with the C_6v_ symmetry. (b) Nontrivial unit cell with the C_3v_ symmetry and corresponding band diagrams and eigen field distributions.

# Trivial and nontrivial unit cells without via holes

Investigating whether we can design a similar topological impedance surface in the middle layer without via holes is an important idea. As shown in the eigenmode simulation in Fig. S2, we observe that the opening of Dirac degeneracy at valley points is achieved by breaking the C_6v_ symmetry to C_3v_ symmetry. This process is consistent in unit cells both with and without via holes shown in Fig. S1.

However, if we remove the via holes, numerous waveguide modes appear. The via holes along the z-axis are essential for suppressing TE waveguide modes because they prevent any electric vector from being parallel to the metallic via holes. We further establish the full simulation model without via holes in CST Microwave Studio, as shown in Fig. S3. The topological edge transmission can still be revealed clearly; however, the field distribution can see the overlap of TE waveguide modes. It is proved that via holes of the unit cell can suppress the TE waveguide modes, which is essential to generate a pure topological edge mode.


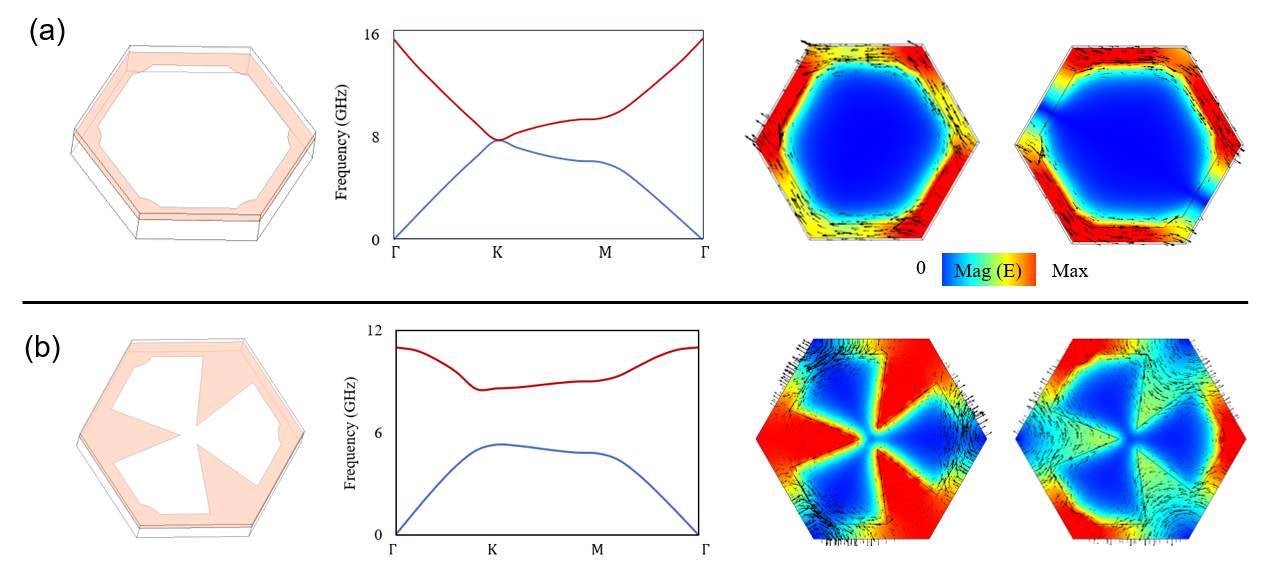


**FIG. S2. Trivial and nontrivial unit cells without via holes.** (a) Trivial unit cell with the C_6v_ symmetry. (b) Nontrivial unit cell with the C_3v_ symmetry and corresponding band diagrams and eigen field distributions.


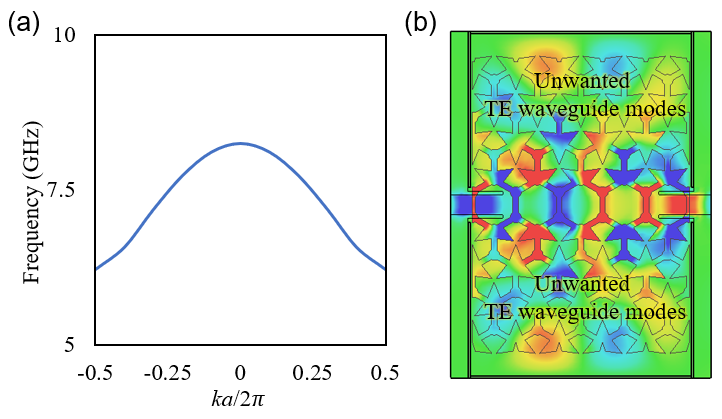


**FIG. S3. Edge states in the structure without via holes.** (a) Dispersion of the edge state. (b) Field distributions in the even-mode TPSIW without via holes.

# Parameters affecting topological edge states

The size of the triangular patches determines the transmission band of the topological edge states. As shown in Fig. S4, by tuning the size of the patches, we can adjust the passband, which is evident from the dispersion curves. When the size of the triangular patches decreases, the passbands of both even and odd TP modes shift towards a higher frequency band.

The odd mode maintains a nearly constant bandwidth of approximately 1.5 GHz, meaning that both the high and low cut-off frequencies will shift simultaneously. The low cut-off frequency of the even mode is fixed around 7.25 GHz, while the high cut-off frequency is determined by the size of the triangular patches, shifting from 8.5 GHz to 9.5 GHz. We also simulated the even-mode TPSIW as an example and obtained S_21_ curves shown in Fig. S4(d). The high cut-off frequency (*f*_ch_) shifts in the same manner as indicated by the dispersion curves.


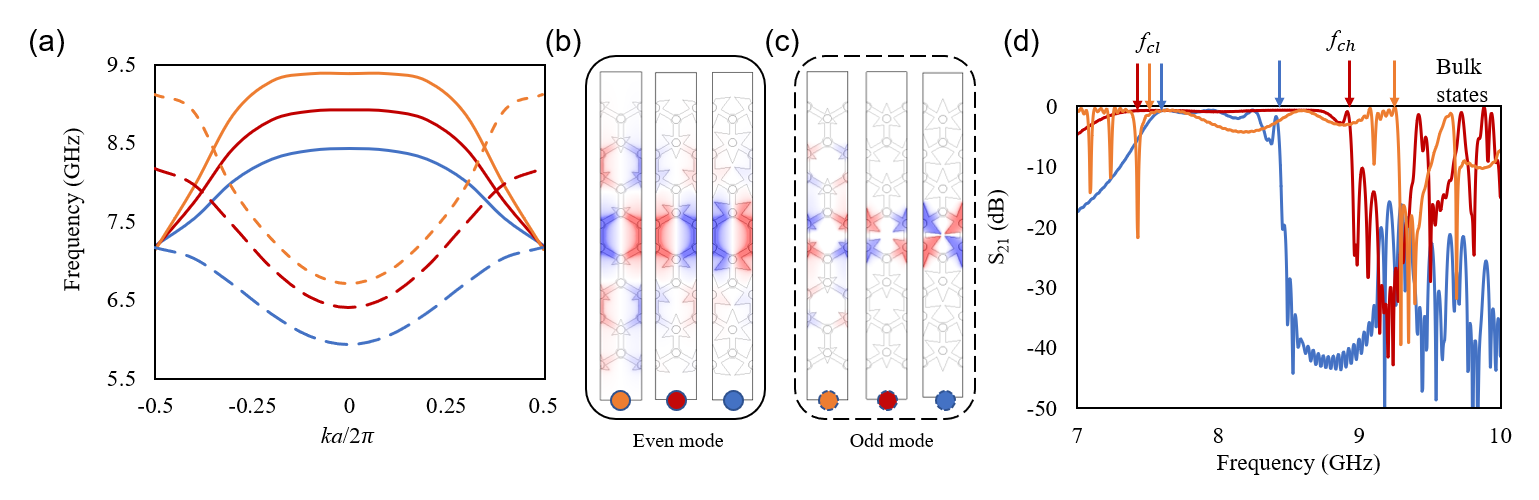


**FIG. S4. Parameter sweeping of the size of triangular patch**: side length is 4.54 mm (blue), side length is 3.41 mm (red), side length is 2.27 mm (orange). (a) Edge state dispersion curves. (b) Electric field distributions of the even mode. (c) Electric field distributions of the odd mode. (d) S_21_ curves corresponding to even-mode TP dispersion curves shown in (a).

The superlattice of an ideal valley topological edge is shown in Fig. S5(a). In order to optimize the terminal matching performance of the even-mode TPSIW, we modify the center patch as shown in Fig. S5(b) and further remove the connection lines when designing TPLWA as shown in Fig. S5(c). To some extent, the structures in Fig. S5(b) and (c) are topological waveguides with line defects. In order to confirm the consistency of topological states, we conduct eigen-mode analysis of the superlattices, and the calculated dispersion curves of three structures are presented in Fig. S5(d). We confirm that even-TP mode always exists on different interfaces with or without defects.

# Optimization of the matching performance

The matching efficacy is notably contingent upon the geometric parameters of the slots at both terminals, as depicted in the parameter sweeping analysis in Fig. S6. For instance, a noteworthy impact on the matching performance of the even-mode TPSIW is observed when adjusting the depth of the slot ($g_{1}$) from 5 mm to 11 mm, as shown in Fig. S6(a)-(c). In the odd-mode TPSIW design, the width of the slot ($g_{5}$) can even affect the transmission bandwidth, as shown in Fig. S6(d)-(f).


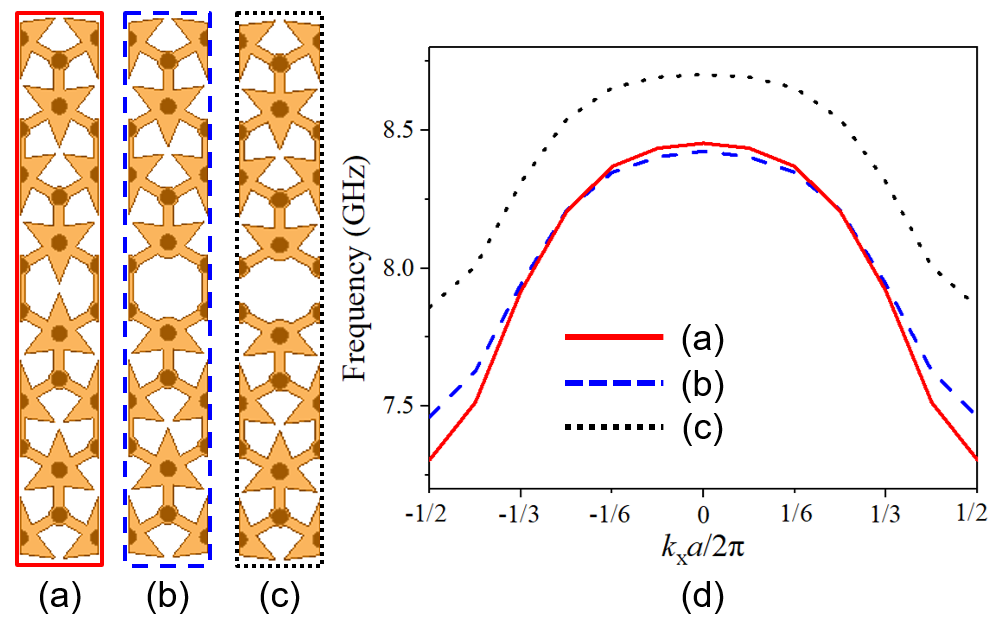


**FIG. S5. Comparison of even-TP modes without or with defects.** (a) Ideal topological interface. (b) Interface with a defect by removing the pair of triangular corner patch. (c) Interface with a defect by removing the central connecting lines. (d) Dispersion curves of three different interfaces.

Moreover, via holes surrounding the feeding structure is also important to tune the matching performance. We have added some simulation models with different via holes to show the influence. As shown in the Fig. S7, if we delete only one pair of via holes surrounding the feeding line, the matching performance will be affected severely as revealed by simulated S parameters.


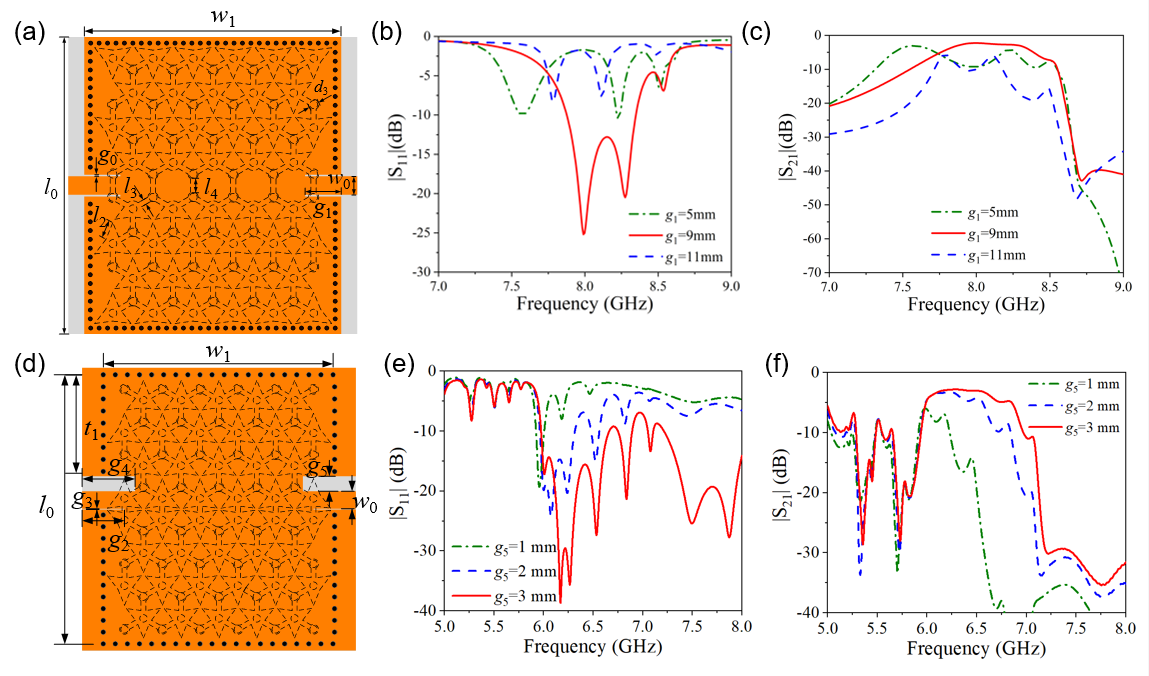


**FIG. S6. Parameter optimization based on simulations.** (a)-(c) Slot depth affect the even-mode TPSIW. (d)-(f) Slot width affect the odd-mode TPSIW.


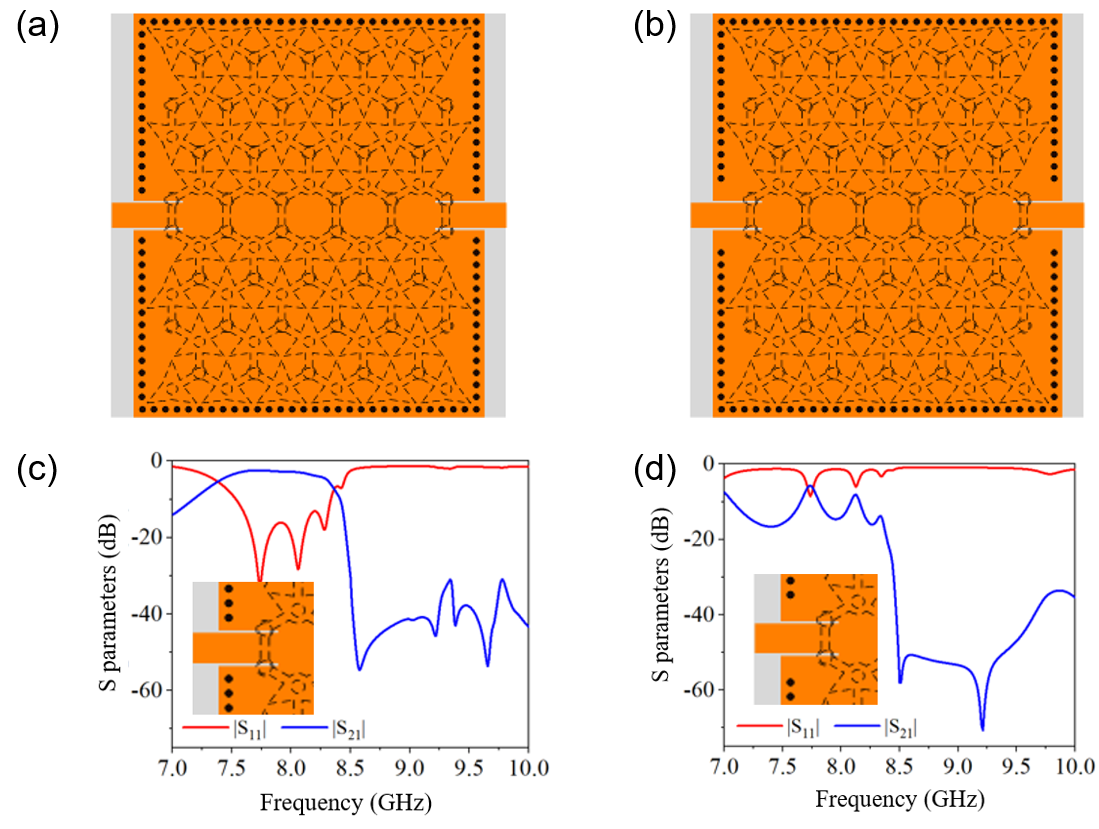


**FIG. S7. Influence of the via holes surrounding the feeding line.** (a) Optimized even-mode TPSIW. (b) Even-mode TPSIW with a pair of deleted via holes around the feeding line. (c) S parameters of the in (a). (d) S parameters of the TPSIW in (b).

# Robustness against PEC defects

The design is compatible with multi-layer PCB processes. We discuss the robustness of the valley photonic crystal, as shown in Fig. S8. We analyze Z-edge waveguides with different defects. It is found that when the height of PEC defect is shorter than those via holes (< 1.524 mm), the waveguide shows strong robustness against large-scale defects even if the defect is located at the interface. However, when the defects are higher than those via holes (>1.524 mm), the transmission of waveguide is affected severely and topological edge states no longer exist.


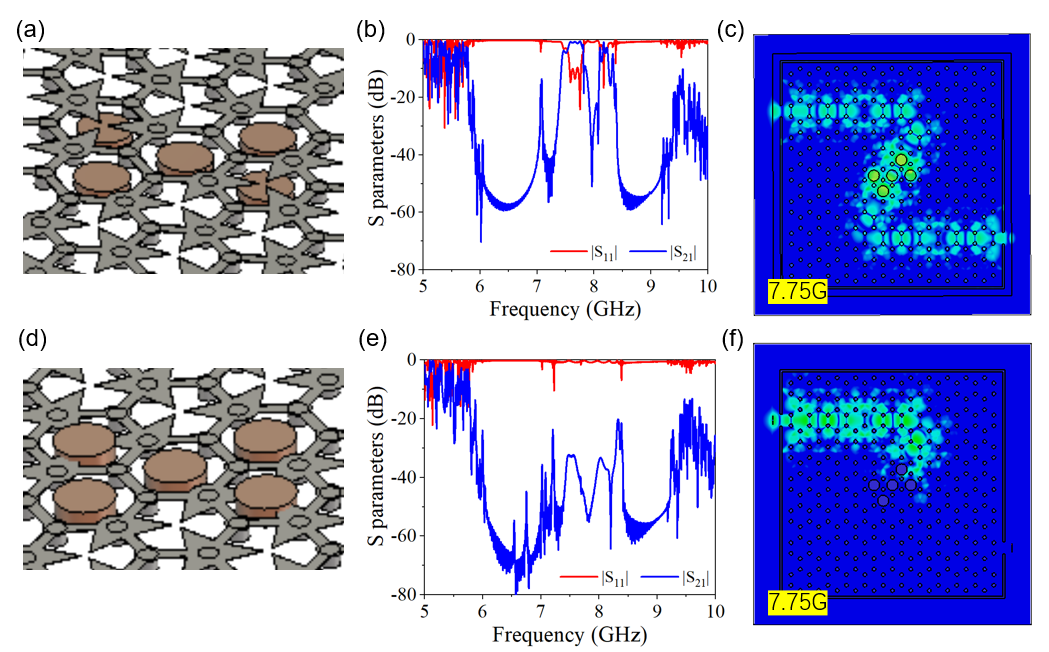


**FIG. S8. Defects consisting of five PEC rods at the interface.** (a) The geometric parameters of the short PEC rods are as follows: Radius is 3mm, height is 1mm. (b) Corresponding S parameters. (c) Robust transmission through the defects. (d) The geometric parameters of the high PEC rods are as follows: Radius is 3mm, height is 2 mm. (e) Corresponding S parameters. (f) Total reflection at the defects.

# Uniform odd-mode TPLWAs

We also present the design of uniform TPLWAs using both even and odd TP modes. Through simulations, we conclude that the even TP mode cannot generate efficient radiations from the uniform slot, while the odd TP mode can be radiated from a pair of uniform slots. The even TP mode is predominantly confined to the central interface with symmetrically distributed electric fields on both sides and synchronously phased. It seems intuitive to create a longitudinal slot along the midpoint of the even-mode TPSIW, as shown in Fig. S9(a); however, this structure alone does not generate leaky-wave radiation because the tangential component of the electric vector at the TPSIW slot manifests an anti-phase, causing a cancellation of radiation, evident in Fig. S9(b).

Conversely, the odd TP mode is confined on both sides of the interface, characterized by an anti-phase property, resulting in the electric energy zero point at the interface (as shown in Figure 1(e)). To harness the radiation capabilities, we implement two uniform longitudinally positioned slots on the top of the odd-mode TPSIW, precisely at the nearest unit cell centers on both sides of the interface, as shown in Fig. S9(c). Leveraging the anti-phase nature of the odd TP mode, the electric vectors across the two slots synchronize, culminating in the radiation of the two slots merging into a single summed beam in the far field, elucidated in Fig. S9(d). The simulated beam scanning properties, as illustrated in Fig. S9(e) and (f), demonstrate forward beam scanning characteristics attributed to the forward dispersion of the odd TP mode. Notably, the beam scanning associated with the odd TP mode does not extend across the broadside, necessitating further resolution through the design of periodic TPLWAs.


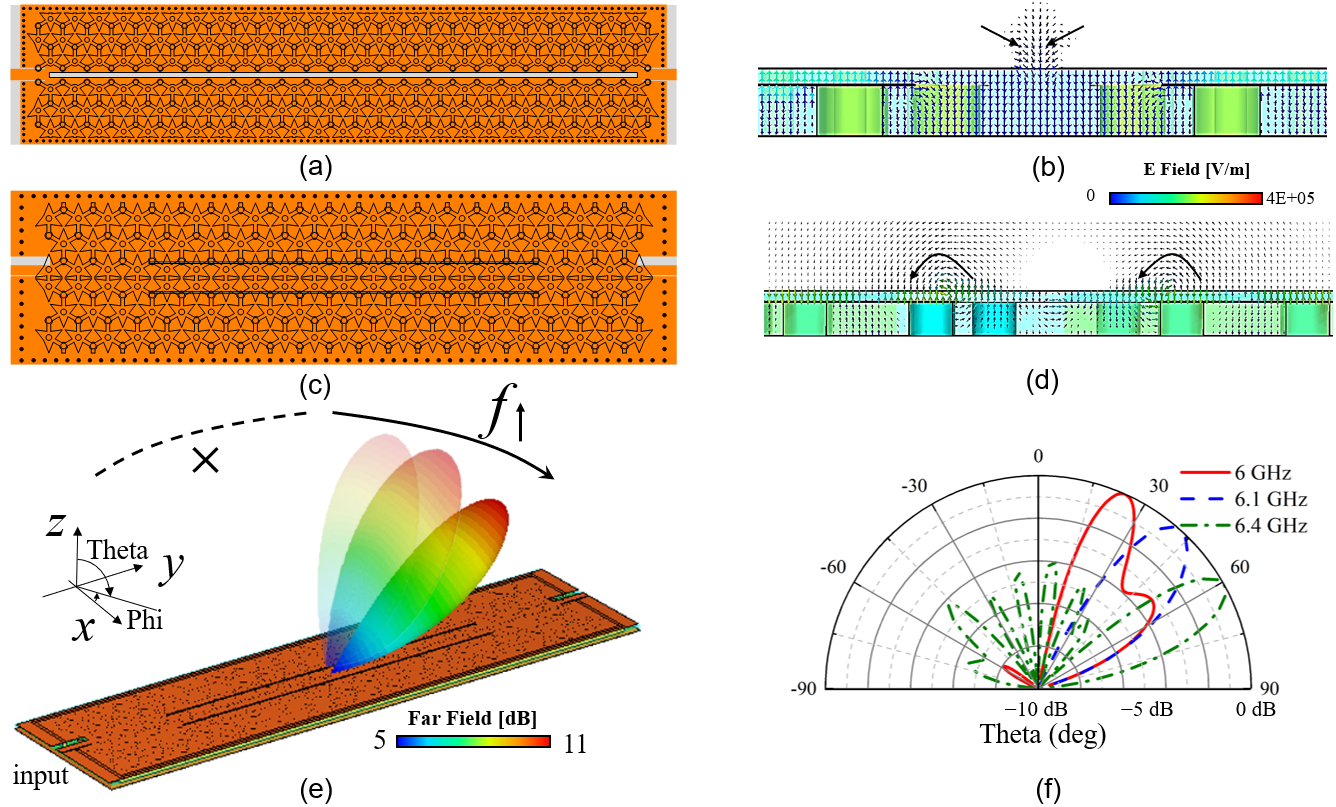


**Fig. S9. Uniform TPLWA.** **a,** Even-mode uniform TPLWA with one slot along the midpoint. **b,** Cross section of electric field vector distribution at the slot with the anti-phase property across the slot, and the radiation is cancelled with each other. **c,** Odd-mode uniform TPLWA structure with two uniform slots. **d,** Cross section of the electric field vector distribution at the slots whose transverse electric field is on phase to generate radiation. Forward beam scanning of the odd-mode uniform TPLWA in **e,** 3D free space and **f,** 2D cross section.

# Transform of electric vector distributions of even-mode TPLWAs

Fig. S10 directly reveals the radiation mechanism of even-mode TPLWAs. We analyze the influence of loaded through vias, which connect the top layer and bottom layer, and the influence of the shape of slots. We numbered the different structures from Ⅰ to Ⅲ, representing to straight slot, straight slot with periodic through vias (red dots), and periodic sinusoidal slot with periodic through vias (red dots). We also analyze field distributions at different cross sections, which are numbered from 1 to 4. As we can see that the electric vector around the straight slot is always anti-phase whose radiation will be cancelled with each other, so we can not realize radiation by cutting a straight slot on the top of the even-mode TPSIW. When adding periodic through vias (4 vias as a unit of P_m_ period), we can obtain transverse electric vectors at the position of through vias, which can show radiation ability; however, most other cross sections still lack radiation ability. We further design the periodic sinusoidal slot, the electric vectors at any cross section become transverse with the radiation ability. In conclusion, the loaded through vias and the periodic sinusoidal slot are the two key elements to realize leaky wave radiation.


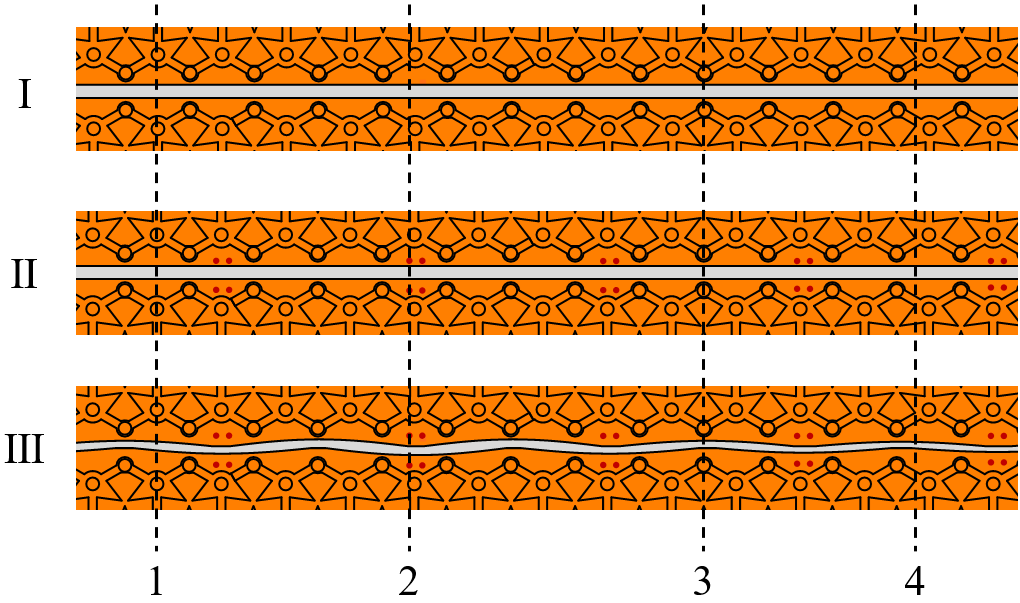


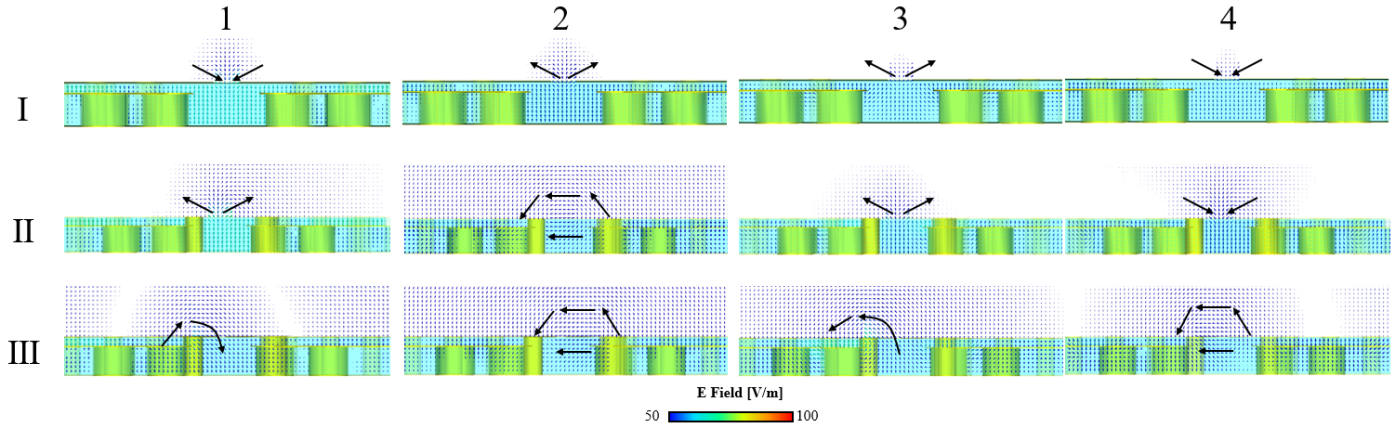


**FIG. S10. Varying electric field distributions of even-mode TPLWAs with different slot designs.** Different structures: Ⅰ: straight slot, Ⅱ: straight slots with periodic through vias, and Ⅲ: the sinusoidal slot with periodic through vias. Electric field distributions at four different cross sections: from 1 to 4.

# Radiation efficiency of even-mode TPLWAs

We simulated the lossy antenna model where the S parameters are applied to obtain the total attenuation constant curve: $P_{out}=P_{in}\times exp(-2\int_{0}^{x} \alpha_{t}(\tau)d\tau)$, as shown in Fig. S11.


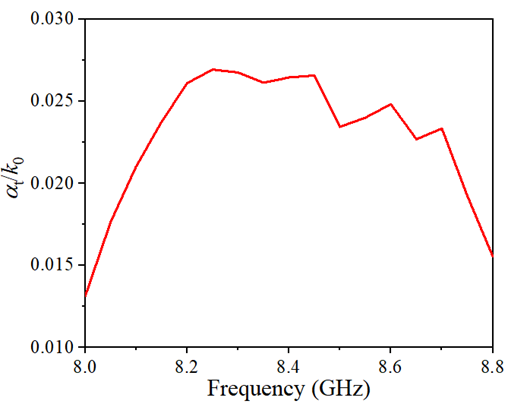


**FIG. S11. Total attenuation constant extracted from S parameters.**

We further set loss and radiation monitors at discrete frequencies to analyze radiation, loss in metals, and loss in the dielectric substrate, separately. Therefore, the total attenuation constant can be divided as three different parts, as shown in Fig. S12. It is obvious that the dielectric loss from substrate and the radiation are two main parts of the attenuation constant. Therefore, the loss of TPSIW limits the radiation efficiency.

**
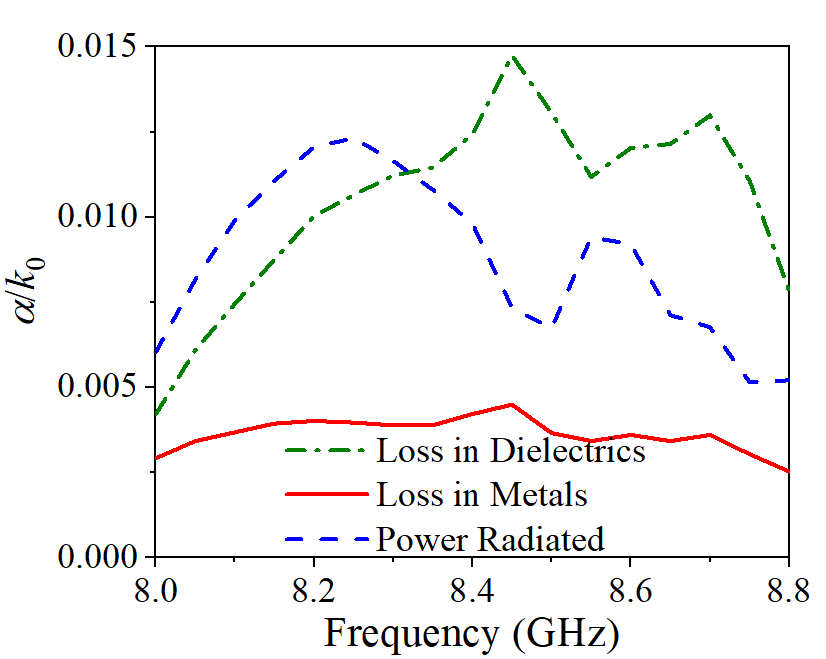
**

**FIG. S12. Different attenuation constant curves extracted from field monitors.**

Total radiation efficiency is plotted in Fig. S13, the radiation efficiency of lossy model cannot exceed 45%; however, if we consider an ideal lossless model, the radiation efficiency of the same antenna will increase to over 90%. Note that the total radiation efficiency curve shows a minimum point at 8.5 GHz, which is caused by the open-stopband phenomenon corresponding to the broadside radiation (Theta = 0 deg). In the following work, we will consider the utilization of a lower loss dielectric substrate (such as the air-filled SIW structure in the reference) as well as the open-stopband suppression technology.


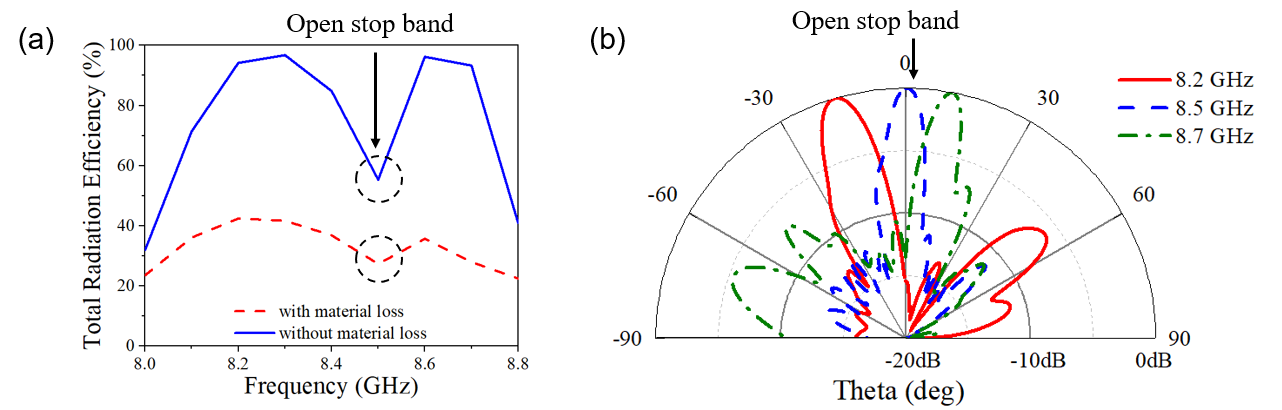


**FIG. S13. Total radiation efficiency and open-stopband phenomenon.** (a) Total radiation efficiency with and without loss. (b) Radiation pattern.

# Multi-channel wireless link based on TPLWA

The transmitter (TX) and receiver (RX) are based on the FPGA (ZYNQ XC7020) and a highly integrated radio frequency (RF) agile transceiver chip (AD 9361). We use a mixer to ensure the center frequency of wireless signals are set at the working frequencies of the TPLWA. We show the Simulink configurations of the TX and RX in Fig. S14.


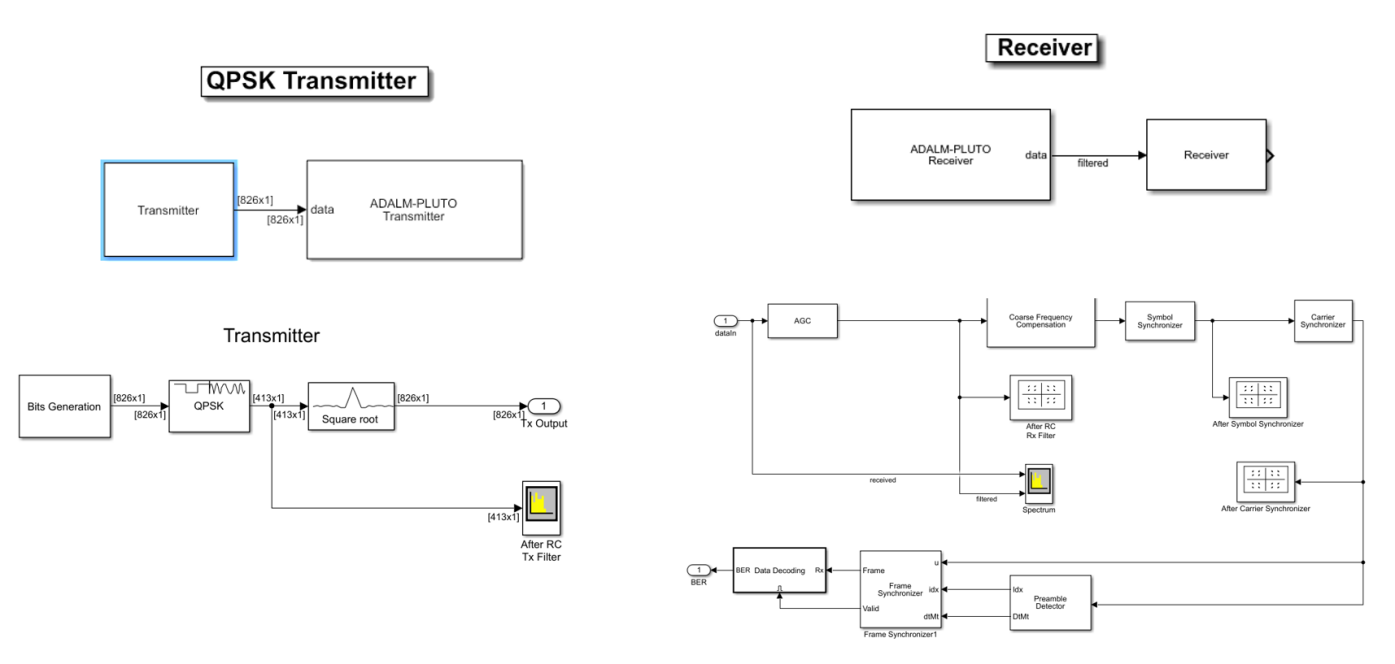


**FIG. S14. TX and RX SDR configurations defined by SIMULINK.**
